# Complete genome sequence of the lumpy skin disease virus reported from Jammu and Kashmir, India, during 2022 outbreak

**DOI:** 10.1128/MRA.00317-23

**Published:** 2023-10-31

**Authors:** Shabir Bhat, Waseem Chaudry, Purnima Mittal, Bilal Ahmad, Zahoor Haroon, Shaista Shaheen, Masood Mir, Manjunath Reddy, Mohammad Wani

**Affiliations:** 1 Animal Husbandry Department Kashmir, Institute of Animal Health and Biological Products, Zakura, Srinagar, Jammu and Kashmir, India; 2 Directorate of Animal Husbandry Kashmir, Srinagar, Jammu and Kashmir, India; 3 National Institute of Veterinary Epidemiology and Disease Informatics, Bengaluru, Karnataka, India; Portland State University, Portland, Oregon, USA

**Keywords:** Complete genome sequence, Lumpy skin disease virus

## Abstract

Lumpy skin disease virus (LSDV) is the causative agent of an economically important disease of cattle and water buffaloes. Here, we announce the complete genome sequence of the LSDV from Jammu and Kashmir, India. LSDV/02/KASH/IND/2022 was detected in skin biopsy sample of an LSD-infected dairy cow on 24 October 2022.

## ANNOUNCEMENT

Lumpy skin disease (LSD), a highly contagious, vector-borne viral disease of cattle and water buffaloes, is caused by the lumpy skin disease virus, a member of genus *Capripoxvirus* within the Poxviridae family (1). In India, LSD was reported in August 2019 (2 – 4). Here, we announce the complete genome sequence of the lumpy skin disease virus (LSDV/02/KASH/IND/2022) detected from an LSD outbreak in Jammu and Kashmir. On 24 October 2022, at Kapran, Anantnag, Jammu and Kashmir, a skin lesion biopsy sample was taken from a dairy cow showing clinical signs consistent with LSDV infection. The LSDV DNA was isolated from a skin lesion biopsy specimen using a DNeasy blood and tissue mini kit (QIAGEN, Germany) according to the manufacturer’s instructions. DNA was mechanically sheared into smaller fragments by Covaris. The paired-end sequencing library was prepared using the QIAseq FX DNA Library Kit for Illumina. The average size of libraries was 374 bp. The libraries were analysed on TapeStation 4150 (Agilent Technologies) using High Sensitivity D1000 ScreenTape as per the manufacturer’s instructions. The libraries were sequenced on the Illumina Novaseq 6000 platform (2 × 150 bp chemistry) to generate ~1 GB data/sample. The raw data generated by the sequencer was filtered to remove low-quality bases and adapter sequences using Trimmomatic software version 0.39. The filtered high-quality reads were mapped against the LSDV NI-2490 (NC_003027.1) reference genome using the “Map reads to reference” program of CLC genomics version 22 using default parameters. Among 14,250,686 total reads, 250,505 (1.76%) were mapped to the reference LSDV genome. Coverage analysis stats with CLC genomics version 22 reflects details of 250,505 mapped reads and 37,786,395 mapped bases for our sample with an average number of reads per base equal to 0.0066.The protein-coding genes were predicted by GATU relative to the LSDV reference genome sequence (NC_003027.1) (5).The reads obtained from the LSDV sample LSDV/02/KASH/IND/2022 were assembled into a double-stranded, linear DNA contiguous sequence of 150,611 bp, with an evenly distributed average GC content of 25.9% (6). LSDV/02/KASH/IND/2022 contains a 147,911-bp central coding region ﬂanked by two inverted terminal repeats of 1429 bp. The LSDV/02/KASH/IND/2022 sample shares 99.75% identity with LSDV reference genome using MEGA-X. A total of 177 variants were identified including 145 single-nucleotide variants, and 32 small indels were identiﬁed when compared to LSDV reference genome using CLC genomics version 22. Some of these mutations resulted in amino acid changes in the proteins (Table 1). There is deletion of four amino acids (L_32_, S_33_, T_34_, and I_35_) in the G protein-coupled chemokine receptor (GPCR, WHU51624.1). The amino acid sequences of GPCR gene (LSDV010) of our sample contained only two unique signatures of LSDV (A_11_ and T_12_) (7).

**TABLE 1:** Amino acid changes within the genome of LSDV/02/KASH/IND/2022 in comparison to reference genome (NC_003027.1)

S.no	ORF	CDS	Amino acid changes	Protein_id
1.	LSDV005	2891..3586	E192K	WHU51619.1
2.	LSDV010	6896..8029	27–30 del LSTI	WHU51624.1
3.	LSDV011	8135..8770	N71K & S113F	WHU51625.1
4.	LSDV024	18853..20769	K190E	WHU51638.1
5.	LSDV032	27891..28496	T14N & P98S	WHU51646.1
6.	LSDV064	57306..57899	172 del D	WHU51678.1
7.	LSDV070	64318..64890	R73K	WHU51684.1
8.	LSDV075	65885..68281	L599S	WHU51686.1
9.	LSDV089	83143..84006	A225V	WHU51699.1
10.	LSDV087	85723..86175	S28L	WHU51701.1
11.	LSDV094	87136..89121	S82L	WHU51704.1
12.	LSDV096	89772..90281	29 del E	WHU51706.1
13.	LSDV108	100087..101220	A17E	WHU51718.1
14.	LSDV122	116051..116596	E60K and E85K	WHU51736.1
15.	LSDV124	117432..118337	56 ins K and S204F	WHU51738.1
16.	LSDV133	120256..121935	P36L and L382I	WHU51743.1
17.	LSDV134	122090..128167	N327S and K1056R	WHU51744.1
18.	LSDV136	132479..133201	T32M and S52F	WHU51750.1
19.	LSDV137	133250..133924	174 del N	WHU51751.1
20.	LSDV140	135444..137096	D167N, I218N and L547F	WHU51754.1

## Data Availability

The complete genome sequence of LSDV/02/KASH/IND/2022 sample has been submitted in the GenBank under accession number OQ588787.The raw data has been deposited in the SRA database under the BioProject accession number PRJNA932167.
